# The American Dental Convention

**Published:** 1863-08

**Authors:** 


### Editorial.

### THE AMERICAN DENTAL CONVENTION,

Met at Saratoga on the 4th of August. There were over one hundred in attendance. The meeting was one of the most interesting we ever attended. It was replete with interest throughout, but there were some things emphatically so; prominent among which was a description by Dr. N. W. Kingsley, of his method of constructing artificial palates.

The Dr. succeeded in making this very complex and intricate operation, very plain and understandable, a point not always attained, by those who attempt descriptions. The enthusiasm and appreciation of the Convention was manifested by an appropriation of $50 for a gold medal for Dr. Kingsley.

The discussions were good, to the point, and well timed, as will be seen by looking through the pages of this number.

In a review of what was done, we conceive it to be an indication, that this body will go on meeting from year to year with increased energy, vigor and efficiency, and that it will prove itself to be one of the great instrumentalities for professional progress and improvement.

There are two classes of persons, that we think will not see verified their predictions in regard to the Convention. One class has supposed, not long since at least, that the American Dental Association was designed, by its originators and friends, to supercede the Convention, and that all these persons would use their utmost influence to secure that end. So far from this being the case, it is now apparent to all, that many of the most ardent workers in the Association, are abreast with the foremost in the Convention, and are working laboriously to make it efficient and popular.

Another class of persons thought that one or the other of these bodies was or would be superfluous; and that one or the other must necessarily be rooted out. Now it must be patent to every one familiar with the two institutions, that they do not in any respect clash; that the objects and work are quite distinct, and are such as the same persons will to a great extent wish to engage in.

The Association, perhaps from its more definite organic structure, has more of the elements of perpetuity than the Convention.

We shall at all times advocate the employment of every instrumentality that can in any way render aid to our noble profession. T.

